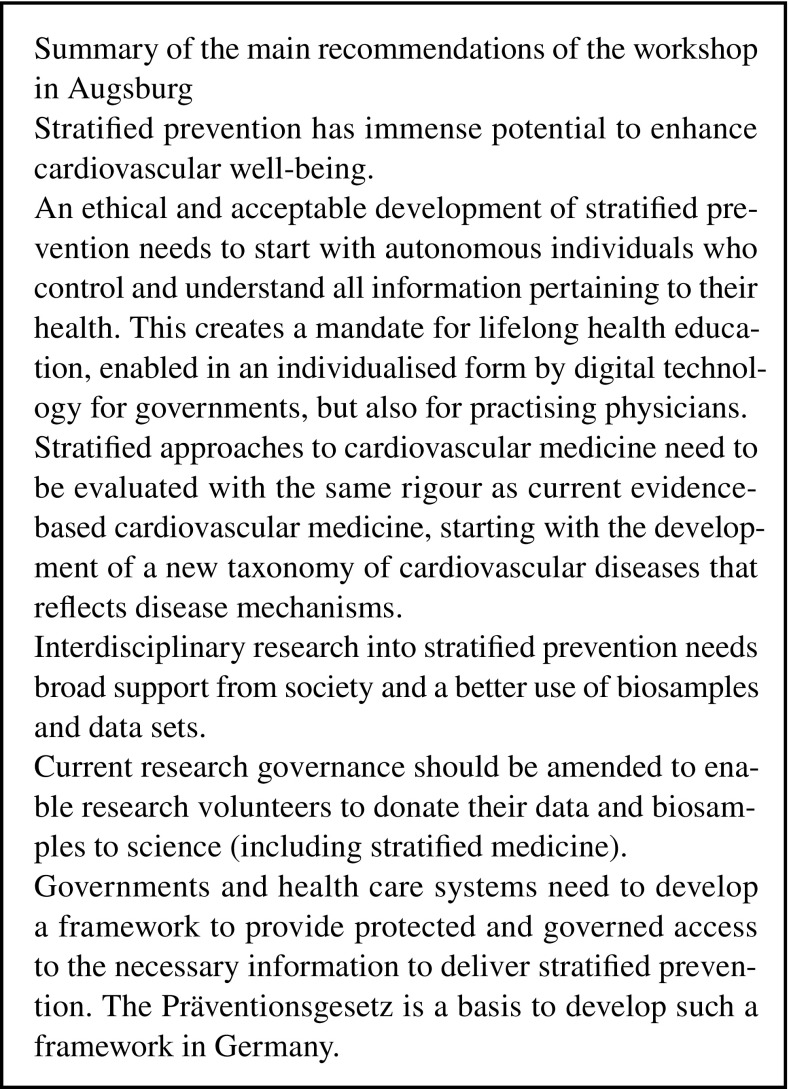# Stratified prevention: opportunities and limitations. Report on the 1st interdisciplinary cardiovascular workshop in Augsburg

**DOI:** 10.1007/s00392-017-1186-y

**Published:** 2017-12-16

**Authors:** Gregor Kirchhof, Josef Franz Lindner, Stephan Achenbach, Klaus Berger, Stefan Blankenberg, Heiner Fangerau, Henner Gimpel, Ulrich M. Gassner, Jens Kersten, Dorothea Magnus, Herbert Rebscher, Heribert Schunkert, Stephan Rixen, Paulus Kirchhof

**Affiliations:** 1 University of Augsburg, Augsburg, Germany; 2 University Hospital Erlangen, Erlangen, Germany; 3 University of Münster, Münster, Germany; 4 Universitäres Herzzentrum Hamburg-Eppendorf, Hamburg, Germany; 5 University Hospital Düsseldorf, Düsseldorf, Germany; 6 Ludwig Maximilian University Munich, Munich, Germany; 7 University of Hamburg, Hamburg, Germany; 8 Hamburg, Germany; 9 University of Bayreuth, Bayreuth, Germany; 10 University of Birmingham, Birmingham, UK

**Keywords:** Genomics, Prevention, Heart failure, Atrial fibrillation, Stratified medicine, Personalised medicine, Payor, Health economics

## Abstract

Sufficient exercise and sleep, a balanced diet, moderate alcohol consumption and a good approach to handle stress have been known as lifestyles that protect health and longevity since the Middle Age. This traditional prevention quintet, turned into a sextet by smoking cessation, has been the basis of the “preventive personality” that formed in the twentieth century. Recent analyses of big data sets including genomic and physiological measurements have unleashed novel opportunities to estimate individual health risks with unprecedented accuracy, allowing to target preventive interventions to persons at high risk and at the same time to spare those in whom preventive measures may not be needed or even be harmful. To fully grasp these opportunities for modern preventive medicine, the established healthy life styles require supplementation by stratified prevention. The opportunities of these developments for life and health contrast with justified concerns: A “surveillance society”, able to predict individual behaviour based on big data, threatens individual freedom and jeopardises equality. Social insurance law and the new German Disease Prevention Act (Präventionsgesetz) rightly stress the need for research to underpin stratified prevention which is accessible to all, ethical, effective, and evidence based. An ethical and acceptable development of stratified prevention needs to start with autonomous individuals who control and understand all information pertaining to their health. This creates a mandate for lifelong health education, enabled in an individualised form by digital technology. Stratified prevention furthermore requires the evidence-based development of a new taxonomy of cardiovascular diseases that reflects disease mechanisms. Such interdisciplinary research needs broad support from society and a better use of biosamples and data sets within an updated research governance framework.

## From the “sextet of prevention” to the development of stratified prevention

Interactions between genetic susceptibility, acquired behaviour, formative life events and environmental factors determine the development of several chronic diseases, including cardiovascular conditions. The increasing availability of large data sets that integrate genomic information, specific organ function, and behavioural patterns in populations allows to precisely estimate individual risks of common diseases and their drivers more precisely. Such analyses enable individualised treatment of patients and a stratification of preventive interventions [1, 2]. Stratified prevention has tremendous potential for the prevention of cardiovascular diseases such as coronary heart disease, heart failure or atrial fibrillation, which develop slowly and chronically. The starting point of stratified prevention is the implementation of tried-and-tested truths: healthy life styles, described since the Middle Ages and later supplemented by smoking cessation, are supported by a large evidence base (textbox) [3].


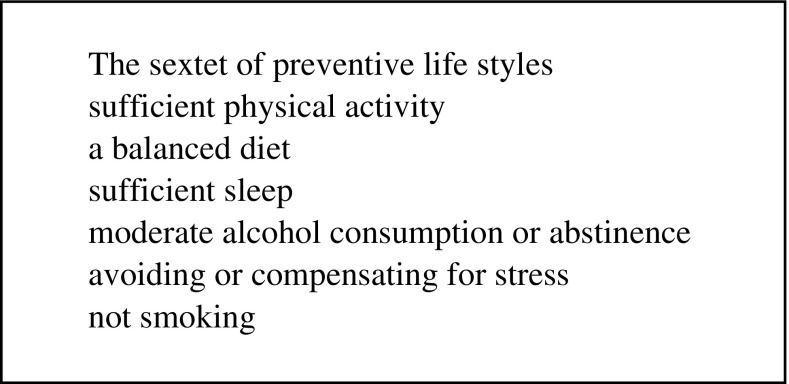


Society and governments have a duty to support implementation of healthy life styles and to provide a healthy environment, e.g. clean water, sufficient nutrition, access to essential vaccinations, screening visits for children and pregnant women, and possibly a mandate to provide access to clean air. There is an undisputed requirement to provide such a healthy environment. In addition, adequate information about the impact of everyday decisions on health is within the remit of a democratic society, e.g. as written and colour-coded warnings on foodstuffs [4]. A free and democratic society also limits the power of the state to subconsciously encourage healthy behaviour without enacting provisions of regulatory law (“nudging”) [5]. While “nudging” may help to encourage decisions and behaviour that protects health, such interventions are only acceptable when they are planned and conducted transparently by a democratic state, respecting citizens’ rights and freedom [5]. As such, legal stipulations to locate sweets and alcohol in supermarkets in places that disincentivise their purchase may be acceptable. Exaggerations that are not in line with the rule of law would be subliminal advertising or other manipulative activities by public authorities promoting a healthier lifestyle, or the historical example—under administrative law—of the “health confession” proposed by Leibniz [6, 7].

## The “preventive self”

Improved hygiene and nutrition, the development of antibiotics, and the introduction of immunisation programmes in the nineteenth and twentieth century allowed a quantum leap in life expectancy and a shift in the spectrum of diseases and causes of death (“epidemiological transition”, [8]). Cancer and cardiovascular diseases are now the most common causes of death in industrialised countries. Reflecting this basic change, greater attention is paid to maintaining individual health: society is developing and demanding a “preventive self” [9], and health care provides advanced preventive treatments to prolong life [10–13]. Even though the societal consequences of this trend require further analyses, the goal of a longer, healthier life, and hence also a right to know about potential hazards, is worthy of support. Having said that, knowledge of health risks and preventive interventions to reduce that risk does not create a normative consequence obliging individuals to implement such measures.

Health and disease are a continuum whose extremes are easily defined—fully healthy or definitely ill—while the transitions from “quite healthy” to “slightly unwell” are often more difficult to determine. This has been described over more than 40 years of salutogenesis research, which was triggered amongst other things by the WHO’s broad definition of health. A disease state in this continuum is defined by biological causes of disease and quantifiable disease consequences, but also by the subjective disease experience and its social perception. The simple healthy life style recommendations discussed above (textbox) already identify factors supporting health and risk factors for disease. Encouraging such behaviour will maintain or improve health of an individual. Novel information, e.g. on predisposition to certain diseases, allow a more precise estimation of risks to health. This information further transforms the dichotomy between “healthy” and “ill” to a continuous scale on which people are assigned a calculable risk of physical or mental harm. Knowing one’s own risk or that of others opens up opportunities for personalised prevention, but may also have negative consequences if fear and uncertainty, or a threat of unequal treatment, are the consequence.

## Health information as a central tool to promote prevention in a free society

The implementation of evidence-based life styles (textbox) is often difficult. Barriers to implementation include a lack of motivation to alter behaviour for distant and abstract benefits, a lack of information and education, and adherence to socially encouraged or acquired behaviours and habits encouraged by family, peer group, and the broader social setting. This social environment often maintains or even amplifies unhealthy behaviour [14]. Competing interests also support unhealthy behaviour, e.g. in the form of advertising for consumer goods, and increasingly via digital and “social” media. Everyone has a right to accurate information and knowledge about prevention to enable autonomous, informed decisions on prevention respecting individual rights. Is the resulting information mandate currently satisfied? There seems to be further need for effective and continued information on how to best maintain health by life styles, linked to social consensus that everyone bears responsibility for their own health. The experience to date suggests that suppressing (mis)information (for instance by restricting advertising) is less useful to enable autonomous, informed decisions on prevention than providing accurate information.

The conference of Ministers of Education and Culture adopted a recommendation on “Health education at school” in 1979, and updated it to a “Recommendation on health promotion and disease prevention at school” in 2012. But information about healthy behaviour needs to be a lifelong process, as behaviour is shaped throughout life by cultural and societal factors. Digital technologies (the Internet, smartphones, social media, the digital “surveying of the self”), which are part and parcel of modern everyday life, have the potential to reach populations that are less receptive to current means of health information. In addition, such technology allows a multi-channel architecture of communication incorporating individualised processing, enables context-dependent assistance including integration of bio-feedback from smartphones or fitness trackers [14], and provides feedback on the effects of technology-based information campaigns. Such health information needs to be credible and quality assured, thus calling for continuous update based on novel evidence [14].

## Stratified cardiovascular medicine

**Fig. 1:**
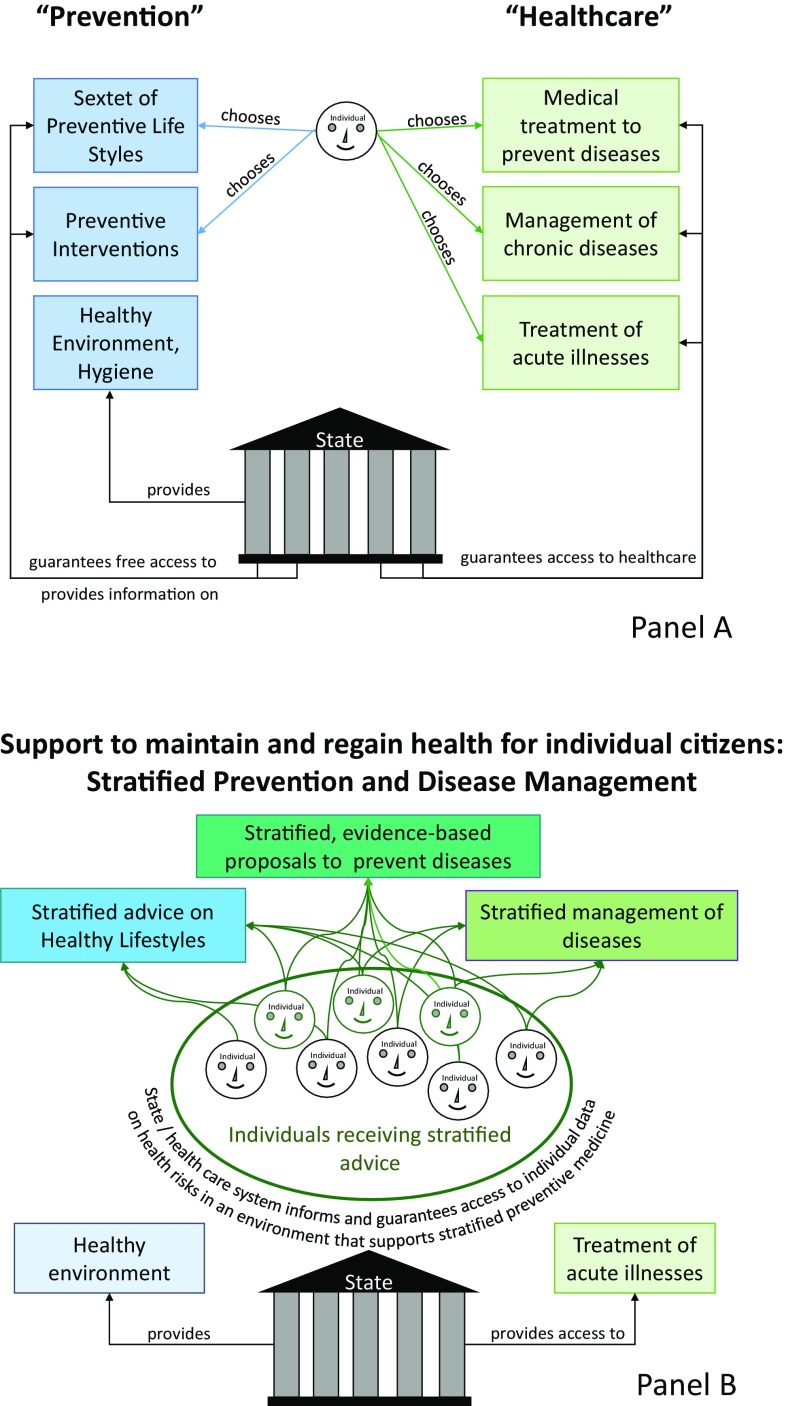
The changing nature of care. **a** Current provision of access to information on healthy lifestyles and access to healthcare. The state provides support to maintain health, e.g. a healthy and safe environment and information about healthy lifestyles. The state also funds (or part-funds) access to healthcare for those who suffer from diseases. **b** Our vision for an interdisciplinary approach to enable stratified prevention and management of health and disease. Here, the state provides stratified support to maintain and regain health, including a secure environment to share information about health and health risks. This environment supports the development and practice stratified prevention and disease management

Prevention and management of cardiovascular diseases has markedly improved in the last decades, relying on evidence-based interventions targeting major mechanisms of heart disease. Consequently, cardiovascular mortality is lower now than it was 20 years ago. Progress has also been made when it comes to describing disease mechanisms and developing and validating targeted therapies of these mechanisms in controlled clinical studies. This is one of the reasons why life expectancy increased in Germany in recent years [15]. One-half of this gain in life years is attributable to cardiovascular prevention, and roughly the other half to improvements in the treatment of patients with cardiovascular diseases, with smooth transitions between prevention and therapy (see Fig. 1, upper panel, for current delivery of cardiovascular prevention and therapy in Germany). These successes have however triggered a “spiral of evidence”, rendering cardiovascular medicine a victim of its own success [15]: ever larger studies are needed to prove the additional benefit of new interventions and treatments, markedly increasing the resources needed to develop new treatments to market. While patient safety justifies such approaches, the effort involved in obtaining approval for new forms of treatment is threatening the development of new cardiovascular therapies [16]. In addition, current disease classifications and regulatory processes incentivise to test and approve new treatments in large patient groups, including those in whom the new therapy does not appear ideal. This lack of appropriate patient stratification is considered as one of the drivers for the “late-phase failures” in the development of cardiovascular medicines [16]. In view of the considerable remaining cardiovascular morbidity and mortality in Europe, these developments need addressing. Particularly, pathophysiological heterogeneity within common cardiovascular diseases has led to a demand for a new disease taxonomy that better reflects disease mechanisms, exemplified for atrial fibrillation in [17]. Disease drivers can be based on genomic and biomedical differences, but also rooted in different social contexts. Describing and classifying these drivers has the potential to unleash stratified prevention and management. This requires flexible thinking and interdisciplinary action in academia, in the cardiovascular drug and device industry, with regulators and with research funders. The European Medicines Agency (EMA) and the Food and Drug Administration (FDA) have already started adjusting the requirements for approval of new medicines. The science-based approach to knowledge generation—proof of the association of characteristics with manifestations of a disease, identification of disease mechanisms and subsequently controlled intervention studies—is still required to validate the safety and effectiveness of such stratified prevention and management approaches.

## The institutional framework for prevention in Germany

Medical prevention is taking place at the crossroads between self-determination and heteronomy, of freedom and obligation to take preventive action. In a free state based on the rule of law, this tension is largely resolved via the fundamental human rights. Individual freedom allows each person to decide whether and how to participate in prevention. State coercion to engage in prevention violates these fundamental rights, and is thus unconstitutional. To protect these fundamental rights, the state also has an obligation to foil coercion imposed by private entities, e.g. insurance companies or employers. Health, on the other hand, has a transcendental significance as it is a prerequisite for a self-determined life. Hence, the state also has a constitutional mandate to promote and protect the health of the individual through information, education and provision of health care for those suffering from disease. These measures can even be required to allow individuals to exercise autonomous prevention, or to decide against it.

A variety of players with their respective specific responsibilities, such as the Federal Centre for Health Education (Bundeszentrale für gesundheitliche Aufklärung, a specialist authority within the remit of the Federal Ministry of Health), the public health service, general practitioners and specialist physicians such as cardiologists, professional organisations, associations and initiatives, as well as the institutions of statutory health, long-term care, accident and pension insurance, seek to perform the constitutional broad educational and information mandate. This reflects the characteristic federal diversity of the German healthcare system. The Disease Prevention Act (PrävG) obliges the social insurance funding institutions within the healthcare system, and the health insurance funds in particular, to develop a “National Prevention Strategy” on which large numbers of agencies collaborate at Federal, Bundesland (federal state), and local authority level. In 2016, the National Prevention Conference adopted for the first time standard national federal framework recommendations spanning funding institutions on health promotion and companies (Fig. 1). The health insurance funds’ recently established Innovation Fund also enables the implementation and evaluation of new prevention methods. Even if in particular the State and the health insurance funds are promoting this development, it is unclear whether better prevention of chronic diseases will reduce or increase costs and expenditure in the healthcare system [18]. At any rate, such measures derive their justification first and foremost from their benefit for the population, and not from economic considerations.

Reflecting on the development of stratified medicine and—unconsciously in some cases—also to evolving concepts for stratified prevention, the legal system then stresses existing research requirements if preventive model projects are planned. Primary prevention, secondary prevention, as well as tertiary prevention, are legally acknowledged in addition to the general promotion of health. Apart from a small number of exceptions such as protective vaccinations, the content of “[p]rimary prevention and health promotion” [the title of section 20 of Book V of the German Social Code (SGB V)] is not legally defined. The concrete measures are defined by individual health insurers (cf. section 20 subsection (1) sentence 1 of Book V of the Social Code). Many projects so far have been pilot projects (“Modellvorhaben”, section 20g of Book V of the Social Code) which aim to generate the required knowledge for effective prevention, including early diagnosis and specific targeting of populations at risk. Unfortunately, most current projects do not have sufficient scientific input and guidance in their design and are underpowered, compromising the generation of credible evidence. For the first time, the Innovation Fund implements an application and evaluation procedure prior to initiation. This may fill the gaps between the current provision of general information and education and the desired targeted prevention of cardiovascular diseases in at-risk populations.

The deep and detailed information required to identify individuals who will benefit from stratified prevention bears the inherent threat of a “brave new world”: access to big data sets could pave the way for a surveillance state that lacks privacy. This may even allow to predict the behaviour of individuals based on analysis of big data sets, opening the door for manipulation by the state or by other interested parties [1, 2]. Furthermore, cumulative restrictions may threaten individual freedom and integrity, e.g. by sanctioning the omission of useful preventive measures or when the right not to know is ignored, which may also promote health. Furthermore, programmes aiming at early diagnosis can lead to misdiagnoses and over treatment with a potential to waste resources and/or to harm. Insights into information identifying specific health risks which form the basis for personalised prevention, e.g. behavioural or genomic data, need to be aligned with the constitutional entitlement to equal rights and obligations for all citizens. State intervention may at times be needed in extremis to maintain equality. The legal starting point and the guiding principle for this harmonisation is the informed, autonomous, free (Fig. 1).

## Legal restrictions and the need to adapt research consent procedures

Better information, the pilot projects discussed in section V, the prevention strategy of the new Disease Prevention Act, and the opportunities and risks of stratified prevention all underpin a broad interdisciplinary research mandate. Ethical research relies on transparent procedures and consent of participating individuals. The subject’s consent to take part in a research project is a central expression of his or her self-determination, which is guaranteed in many ways at the level of fundamental rights and human rights [Art. 2 and Art. 1 of the Basic Law (GG), Art. 3§ 2 of the European Convention on Human Rights (ECHR), Art. 5 of the Convention of the Council of Europe on Human Rights and Biomedicine]. At difference to consent to a medical intervention, the health benefit for the subject of a research project is uncertain. Furthermore, the essence of research is that it is not known in advance what results will be achieved. Moreover, new scientific knowledge may necessitate the re-analysis of existing data for a different research purpose than was originally planned. The advancing research may hence make it appear expedient to re-examine existing data sets or to alter the originally planned course of the research, including the methods that had been considered. This particularly applies today given the furious pace of development in stratified medicine. Such additions to or deviations from the original research plan are however often not covered by current research procedures, and would frequently require once more informing the patient or subject and obtaining their consent, which is almost always impossible, particularly with large volumes of data. What is therefore needed is a new general consent enabling the subject to consent to research as a process, including consent to the re-analysis and re-examination of data and biosamples which were collected during a research project. Such a consent should be comprehensible, targeted and layered. The layering will enable individuals to choose between general or partial consent to the research plans according to a pre-defined scheme, which can make provision for exclusions. This general consent trusts the researchers and research institutions. Hence, research institutions need to provide sufficient governance for ongoing research projects (See "**Governance of stratified research projects and biobanks**"). Participants in a research study are particularly worthy of protection since an individual health benefit is frequently unable to compensate for participation in the test. The protection of the subject and the open-ended nature of the research aims must be reflected in the consent form. The general research regulations (Good Clinical Practice—GCP, International Council for Harmonisation of Technical Requirements for Pharmaceuticals for Human Use—ICH) remain in force. This means

- that the subject is able to consent in general terms to take part in a research project whose aims and goals can only be partially defined at the time of consenting,
- that the re-use of data by third parties requires separate consent, which can be obtained in advance,
- and that information relating to third parties (gene analyses) may only be passed on if the interests of the subjects are taken into account (andwith the consent of all concerned).

Given the general consent to take part in the study and the measures which this entails, oral and written information is to be limited to the essentials of the study, such as the research aims, the general process, the foreseeable risks and the general use of data and biosamples. The consent form needs to enable the subject to understand and consent to the process. Superfluous, overly-detailed information sheets and consent forms are to be avoided here.

## Governance of stratified research projects and biobanks

The undefined outcomes of biomedical research at the outset require a defined governance of research projects overseeing research conduct and communication of findings back to individuals and to the wider research community. Such governance must respect citizens’ right of personality (Art. 2§ 1 of the Basic Law, Art. 7 et seq. of the European Charter on Human Rights and Art. 8 ECHR) and against genetic discrimination (Art. 3§ 1 of the Basic Law and Art. 21 of the European Charter on Human Rights), whilst on the other hand promoting biomedical research (Art. 5§ 3 sentence 1 of the Basic Law and Art. 13 of the European Charter on Human Rights), particularly with a view to individual and public health (Art. 2§ 2 sentence 1 of the Basic Law and Art. 35 of the European Charter on Human Rights). The starting point for the regulation of biobanks in Germany is data protection law [section 2 subsection (2) No. 1 of the Genetic Diagnostics Act (*Gendiagnostikgesetz* GenDG)]. Such “governance”, which is primarily covered by data protection law, however fails to do justice to the requirements of a nuanced research infrastructure: first with respect to the altruistic donations made by many thousands of citizens, and second with a view to their function, namely to promote individual and public health. This calls for a dynamic regulatory approach, which effectively protects the donors’ right of self-determination, guarantees biobank confidentiality, regulates biobanks’ establishment and operation, rights of access and monitoring, as well as providing for rights to refuse to testify under criminal law [18]. The more far-ranging regulatory requirement can be illustrated by taking donors’ right of self-determination as an example: as a matter of principle, the current understanding is that a donation to a biobank must be consented for a specific research project. This however disproportionately restricts the potential for research with regard to future scientific questions which cannot be predicted at the time of consent. A biobank only does justice to its function as a research infrastructure when donations can also be given “for science” as a whole. It is not expedient here to prohibit generalised consent by referring to data protection law. Many participants in research projects and biobanks are motivated by a general wish to “help research”. The general consent corresponding to this wish should be legally enclosed by formal procedures and substantive standards (“governance”). A legislature that accompanies current developments in research can inspire citizens’ legally justified confidence that their generalised consent will be reflected in responsible research. Research sponsors need to develop research governance structures that are effective and research friendly, and which safeguard fundamental rights, in cooperation with ethics commissions, state supervision, and data protection authorities.

## Interdisciplinary research mandate to underpin stratified prevention

It is well established that cardiovascular health can be protected and maintained by getting enough exercise and sleep, a balanced diet, moderate alcohol consumption, not smoking, and suitably dealing with stress (textbox). Research into stratified interventions to widen these healthy behaviours can help to improve cardiovascular health, especially in socially and otherwise defined “areas alienated from prevention”. The law here principally focuses on information to enable the balancing act typifying prevention to be successful: while preserving autonomy of each individual, the mandate to protect and further health should be fulfilled—resulting in a mandate for lifelong health education. Digital technology permits a perhaps decisive step towards easily accessible, targeted, context-independent health information which can then also be evaluated and which could also reach groups of the population which are less willing to take up healthy life styles.

Promotion of healthy lifestyles already blurs the boundary between disease and health, as individuals who are not “ill” are asked to change their behaviour to maintain their health and are placed on a health risk scale. These uncertainties are intensified by stratified prevention: large and deep biomedical datasets and physiological measurements will allow to predict individual health risks in the future, resulting in tailored recommendations for preventive interventions. These insights offer tremendous opportunities to better maintain and protect cardiovascular health in specific individuals at high risk. The stratified nature of such interventions requires adjustments to the paradigm of large clinical trials to evaluate novel interventions, calling for innovative ways to evaluate stratified preventions, similar to the innovative ways that have been outlined for the approval of novel, stratified treatments. As a reaction to the development of stratified medicine and—unconsciously in some instances—also more precise prevention, the legal system rightly stresses the general information mandate, but then also the need for interdisciplinary research, if preventive model projects are provided for. It is uncertain how stratified prevention will affect the cost of health care: the potential to save cost by avoiding chronic diseases may be offset by the cost of treatment across a longer life. As maintaining health and preventing disease is primarily intended to sustainably serve the health of those concerned, the economic impact of personalised prevention can be measured as the concept is being developed.

Stratified prevention offers immense opportunities to prevent diseases in a more effective, lower-risk manner. The necessary interdisciplinary research must devote itself to these elementary goals, but must also address the dangers and fears associated with precise risk prediction. The fears associated with a society formed by predictable individuals are not entirely unfounded, and difficult questions arise regarding equal opportunities or measures which as a rule make sense medically, but which may endanger health in individual cases. The goal is to develop stratified prevention that is ethical, effective, evidence-based and in conformity with fundamental rights, and to make it generally accessible. Interdisciplinary governance of the development of stratified prevention can mitigate these risks to individual freedom while leveraging the potential of stratified prevention for the cardiovascular health of populations and individuals.